# Computational analysis of splicing errors and mutations in human transcripts

**DOI:** 10.1186/1471-2164-9-13

**Published:** 2008-01-14

**Authors:** Yerbol Z Kurmangaliyev, Mikhail S Gelfand

**Affiliations:** 1 Institute for Information Transmission Problems (the Kharkevich Institute) RAS, Bolshoi Karetny pereulok 19, Moscow, 127994, Russia; 2 Faculty of Bioengineering and Bioinformatics, Moscow State University, Vorobievy Gory 1-73, Moscow 119992, Russia

## Abstract

**Background:**

Most retained introns found in human cDNAs generated by high-throughput sequencing projects seem to result from underspliced transcripts, and thus they capture intermediate steps of pre-mRNA splicing. On the other hand, mutations in splice sites cause exon skipping of the respective exon or activation of pre-existing cryptic sites. Both types of events reflect properties of the splicing mechanism.

**Results:**

The retained introns were significantly shorter than constitutive ones, and skipped exons are shorter than exons with cryptic sites. Both donor and acceptor splice sites of retained introns were weaker than splice sites of constitutive introns. The authentic acceptor sites affected by mutations were significantly weaker in exons with activated cryptic sites than in skipped exons. The distance from a mutated splice site to the nearest equivalent site is significantly shorter in cases of activated cryptic sites compared to exon skipping events. The prevalence of retained introns within genes monotonically increased in the 5'-to-3' direction (more retained introns close to the 3'-end), consistent with the model of co-transcriptional splicing. The density of exonic splicing enhancers was higher, and the density of exonic splicing silencers lower in retained introns compared to constitutive ones and in exons with cryptic sites compared to skipped exons.

**Conclusion:**

Thus the analysis of retained introns in human cDNA, exons skipped due to mutations in splice sites and exons with cryptic sites produced results consistent with the intron definition mechanism of splicing of short introns, co-transcriptional splicing, dependence of splicing efficiency on the splice site strength and the density of candidate exonic splicing enhancers and silencers. These results are consistent with other, recently published analyses.

## Background

Vertebrate genes consist of relatively short exons separated by considerably larger introns. The introns of lower eukaryotes, invertebrates and plants are much shorter. This difference may be explained by the preference for two possible mechanisms for recognition of the exon-intron boundaries by the splicing machinery. In the case of long introns, the exon definition mechanism initially recognizes pairs of splicing sites corresponding to one exon. Vice versa, short introns are recognized by the intron definition that pairs splicing sites across introns [1]. Historically, the intron definition mechanism seems to be the ancestral one, whereas exon definition likely is a relatively recent innovation that, in particular, created the possibility of regulated alternative splicing [2].

These models yield different consequences of mutations that destroy splicing sites. Errors in exon definition should lead to exon skipping or, if there are strong cryptic sites, the use of the latter, whereas errors in intron definition should cause intron retention. Indeed, exactly this behavior was observed in vivo and in vitro experiments (reviewed by [1]), and in early analyses of disease-causing mutations of human genes [3,4]. These predictions also agree to the distribution of alternative splicing types in different organisms. In vertebrates, where long introns are frequent, the prevalent type of alternative splicing is exon skipping [5,6], while in plants, where the majority of introns are short, the most frequent type is intron retention [5,7].

Intron retention is the least studied type of alternative and aberrant splicing. In contrast with other types of alternative splicing, which involve the choice between different splice sites, intron retention represents complete absence of splicing. Some specific features of retained introns have become clear in recent studies of human [8,9] and plant transcriptomes [10]. Retained introns were found to differ from other introns in GC content, that was lower than in exons but higher than in constitutively spliced out introns. Retained introns were shown to be shorter on the average than constitutively spliced out ones and exhibited a tendency to occur in 5'- and 3'-untranslated regions [8-10]; they also have weaker sites [9].

In several cases intron retention clearly has a function. A considerable fraction of retained introns encode identifiable protein domains or parts thereof [8,11]. In some cases intron retention produces different functional isoforms (EBNA-3 family anigens of the Epstein-Barr virus [12]); isoforms with aberrant function (cancerspecific form of cholecystokinin 2 receptor [13]); truncated proteins that may be involved in regulation (cold-dependent lipid metabolism in plants [14], nuclear transport of retroviruses [15], autoregulation of splicing [16]); non-functional proteins (P-element of Drosophila [17] or rat cytochrome P450 *CYP2C11 *in stressed liver [18]); proteins with unknown function (serine protease kallikrein [19,20]); or, finally, isoforms with no known functional differences between the variants (hormone urocortin 1 prepropeptide [21], cyclooxygenase [22], D1 dopamine receptor (DR1) interacting protein calcyon [23], mouse homeodomain transcription factor *Tgif2 *[24]). At that, intron retention may be conserved in vertebrates, e.g. intron 3 of splicing regulator of the SR family *9G8 *[16] or species-specific, e.g. intron 2 of *Tgif2*, present in the mouse gene, but not its human ortholog *Tgif2 *[24].

However, it is likely that many cases of observed intron retention were caused by errors of the splicing machinery. Retained introns are the least conserved type of elementary alternatives [25]. Moreover, large scale projects that aim at sequencing of full-length cDNA use normalization procedures to enrich low copy transcripts, and these procedures seem to increase the fraction of underspliced transcripts that retain one or several introns [26,27]. Traditionally such artifacts in cDNA databases were treated as a nuisance and filtered out in attempts to create "clean" sets of alternative isoforms. We tried to look at introns retained in human cDNA data from another angle, assuming that they capture intermediate states of the splicing process and thus provide a glimpse on the splicing mechanisms.

Another way to look at this mechanism is to analyze consequences of mutations in splice sites. This also has been the subject of several very recent studies. Such mutations have two major possible outcomes: exon skipping and activation of cryptic sites, whereas intron retention is relatively rare [3,28-30]. One of important determinants of the cryptic donor splice site phenotype is the presence of a strong candidate donor splice site in the vicinity of mutated sites [3,31]. Cryptic acceptor splice sites are more frequent in exons than in introns, likely due to depletion of AG dinucleotides upstream of the original acceptor sites [32]. There are differences in the distribution of candidate exonic enhancers and silencers between skipped exons and exons with activated cryptic sites [33].

Here we systematically studied aberrant and mutated splicing. Specifically, we compared lengths of affected and adjacent introns and exons, as well strengths of splice sites and distribution of predicted splicing enhancers and silencers in these and adjacent exons and introns. While confirming many earlier predictions, our study also provides a number of new observations that are largely consistent with existing models of the splicing mechanisms.

## Results

### Comparison of retained and constitutive introns

**Figure 1:**
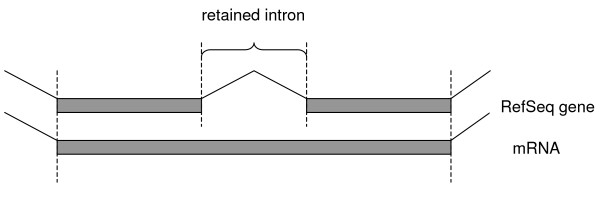
**Definition of scored intron retention events**. Gray rectangles represent exons of the RefSeq gene and mRNA. Exon/intron boundaries are marked by dotted lines.

Sets of retained (Fig. 1) and constitutive (constitutively spliced out) introns were constructed as described in Data and Methods and compared with the aim to identify possible determinants of intron retention. We considered the distribution of intron lengths and of lengths of the flanking exons, scores of intron splice sites and the distal sites in the flanking exons (the acceptor site of the upstream exon and the donor site of the downstream exon), densities of exonic cis-acting elements, intron positions within the gene. The results are summarized in Table 1.

**Table 1:** Properties of retained and constitutive introns. For all intron parameters the medians are reported. The last two columns report the statistical significance of the differences of the distributions by the Kolmogorov-Smirnov test (KS) and Student's t-test (ST); n/s – non significant.

	**introns**			
			
	**Retained**	**Constitutively spliced**	**KS**	**ST**
**Set size**	1197	137580		
**Intron length (nucleotides)**	337	1481	<10^-15^	<10^-15^
**Splice site scores**				
Acceptor site of the of 5'-exon	18,60	19,09	<10^-15^	<10^-11^
Donor site	18,17	18,80	<10^-15^	<10^-15^
Acceptor	18,03	19,06	<10^-15^	<10^-15^
Donor site of 3'-exon	18,74	18,79	n/s	n/s
**Cis-acting elements (candidate sites per nucleotie)**				
ESEfinder: SC35	0,046	0,034	<10^-15^	<10^-15^
ESEfinder: SF2/ASF	0,040	0,028	<10^-15^	<10^-15^
ESEfinder: SRp40	0,041	0,038	<10^-15^	
ESEfinder: SRp55	0,022	0,022	<10^-15^	n/s
RESCUE-ESE	0,050	0,068	<10^-15^	<10^-15^
PESE	0,043	0,035	<10^-15^	<10^-15^
PESS	0,013	0,048	<10^-15^	<10^-15^
**Relative position**				
by ordinal number	0,6	0,5	<10^-15^*	<10^-15^
by gene	0,671	0,597	<10^-9^	<10^-15^
by mRNA	0,446	0,354	<10^-15^	<10^-15^
by mRNA w/o last exon	0,688	0,575	<10^-15^	<10^-15^

* Chi-square test

**Figure 2:**
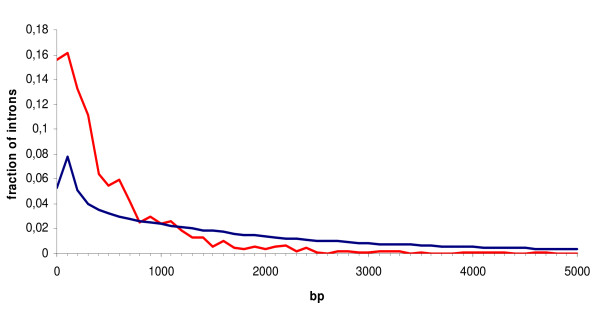
**Histograms of intron lengths**. Red: retained introns; blue: constitutive introns.

The distributions of the intron lengths of retained and constitutive introns were significantly different (Fig. 2, Two-sample Kolmogorov-Smirnov test P < 10^-15^). The retained introns tend to be shorter than constitutively spliced out ones: 84% of the retained introns were shorter than 1000 nucleotides, compared to only 40% of the constitutive introns. The median size of the retained introns was 337, whereas the median size of the constitutive introns was 1481 nucleotides. No significant differences between distributions of flanking exons lengths were observed (data not shown).

Scores of the intron splice sites and splice sites of the flanking exons for retained and constitutively spliced introns were calculated using a positional weight matrix as described in Data and Methods. Splice sites of retained introns were weaker: the distributions of the splice sites scores for the retained and constitutive introns were significantly different for both acceptor and donor sites (Two-sample Kolmogorov-Smirnov test P < 10^-15^). The median scores for the donor sites of the retained and constitutive introns were 18.2 and 18.8 respectively, whereas for the acceptor sites they were 18.03 and 19.06 respectively.

The donor site scores of the 3'-flanking (downstream) exons were similar for the retained and constitutive introns, whereas the acceptor sites of the 5'-flanking (upstream) exons were considerable weaker for the retained introns compared to the constitutive ones, with medians 18.6 and 19.1, respectively (Two-sample Kolmogorov-Smirnov test P < 10^-10^).

**Figure 3:**
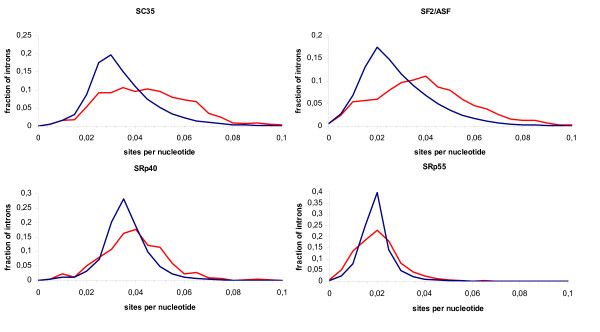
**Histograms of ESE densities predicted by ESEfinder**. Red: retained introns; blue: constitutive introns.

**Figure 4:**
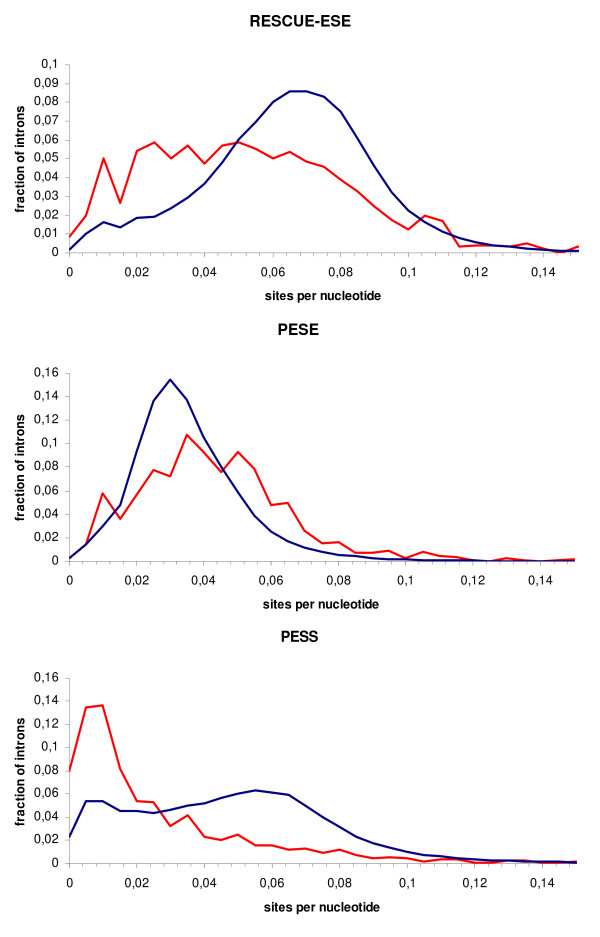
**Histograms of ESE densities predicted by RESCUE-ESE and PESX/PESE and ESS densities predicted by PESX/PESS**. Red: retained introns; blue: constitutive introns.

Densities of cis-acting elements of both types of introns were calculated using three available programs, ESEfinder [34], RESCUE-ESE [35], and PESX [36,37], as described in Data and Methods. The results are described in Table 1. The densities of most types of predicted exonic splicing enhancers (ESEs) were higher in the retained introns, whereas the density of exonic splicing silencers (ESSs) was higher in the constitutive introns (Fig. 3, 4).

At that, the average densities of all four ESEfinder motifs were higher in the retained introns (Fig. 3). The maximal difference between the median densities were observed for the SF2/ASF sites (median densities 0.040 and 0.028 for the retained and constitutive introns, respectively), whereas the lowest difference was observed for the SRp55 sites (median densities 0.0217 and 0.0215, non-significant). The density of PESE octamers (enhancers) was also higher in the retained introns (Fig. 4), whereas the density of PESS octamers (silencers) was higher in the constitutive introns (Fig. 4). In contrast, the density of ESE hexamers predicted by RESCUE-ESE was significantly higher in the constitutively splice introns than in the retained ones (Fig. 4). All these differences were statistically significant (Two-sample Kolmogorov-Smirnov test P < 10^-15^).

The relative position of an intron in a gene was defined as the ratio RP = D/L, where D was the distance from the gene 5'-end to the intron 5'-end (the donor site), and L was the gene length (the distance between 5'- and 3'-ends, as listed in RefSeq). Since terminal exons and introns may have considerably different lengths ([38], and data not shown), the distances were calculated in several different settings. Firstly, we used unspliced genes, as annotated in RefSeq, and in this cases the distances were calculated using the genomic sequence. Secondly, we considered spliced genes: all introns were removed and the studied intron was reduced to a single point, "intron shadow", and the distances were calculated using the mRNA sequence. Thirdly, we considered spliced genes with the last exon removed as well. Finally, we defined relative position of an intron as its ordinal number divided by the total number of introns in a gene.

**Figure 5:**
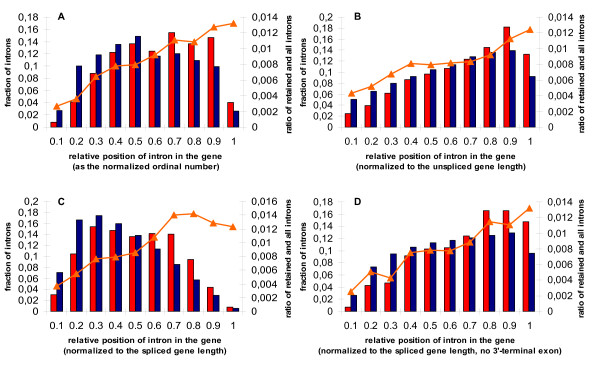
**Histograms of the relative intron positions**. A: the relative (ordinal) intron number; B: unspliced genes; C: spliced genes; D: spliced genes with the last exon removed (see the text for the detailed explanation). Left axis: the fraction of introns in each position bin is given for retained (red) and constitutive (blue) introns separately. Points 0 and 1 on the horizontal axis correspond to the 5'- and 3'-ends of the gene, respectively. Right vertical axis and the orange triangle curve: the fraction of retained introns among all introns in the bin.

The constitutive introns (blue bars in Fig. 5) are shifted towards the 3'-end in the unspliced gene calculations (Fig. 5b), and towards 5'-ends in spliced gene calculations (Fig. 5c). This is consistent with decreasing intron density and increasing exon length in the 5'-to-3' direction [38]. Indeed, when the last 3'terminal intron is removed, the distribution becomes almost uniform (Fig. 5d).

The situation with retained introns is dramatically different (Two-sample Kolmogorov-Smirnov test P < 10^-15 ^for relative intron positions in case with spliced genes and spliced genes with the last exon removed, and P < 10^-9 ^for unspliced genes; the χ^2^-test P < 10^-15 ^for the ordinal intron number). The distribution of the retained introns (red bars in Fig. 5) is considerably shifted towards the 3' in all settings, as compared to the constitutive introns. Accordingly, the fraction of retained introns increases in the 5'-to-3' direction, leveling off at about middle of the gene (the orange curve in Fig. 5).

### Comparison of skipped and cryptic-site exons

The sets of splice-site inactivating mutations were collected as described in Data and Methods. Only mutations directly in the donor and acceptor sites were considered. The exons affected by the mutations were divided into skipped exons (S-exons) and exons utilizing cryptic sites (C-exons). The donor and acceptor site mutations were considered both separately and jointly, to increase the statistical power of the observations. The results are summarized in Table 2.

**Table 2:** Properties of skipped exons (S-exons) and exons with cryptic sites (C-exons). For all exon parameters the medians are reported. The last column reports parameters of all internal exons in our dataset of RefSeq genes. MW: the statistical significance of the differences between the S- and C-exons by the Mann-Witney test; n/s – non significant.

	**S-exons**	**C-exons**	**MW**	**Internal exons**
**Set size**				
Mutated donor sites	67	42		
Mutated acceptor sites	42	72		
All	109	114		154846
**Exon length (nucleotides)**				
Mutated donor sites	114	147	0,024	
Mutated acceptor sites	112,5	130	n/s	
All	114	136	0,020	123
**Densities of cis-acting elements(candidate sites per nucleotide)**				
***ESEfinder: SC35***				
Mutated donor sites	0,043	0,042	n/s	
Mutated acceptor sites	0,038	0,045	n/s	
All	0,042	0,043	n/s	0,038
***ESEfinder: SF2/ASF***				
Mutated donor sites	0,025	0,037	0,048	
Mutated acceptor sites	0,036	0,041	n/s	
All	0,028	0,040	0,005	0,036
***ESEfinder: SRp40***				
Mutated donor sites	0,034	0,043	0,006	
Mutated acceptor sites	0,040	0,043	n/s	
All	0,035	0,043	0,004	0,040
***ESEfinder: SRp55***				
Mutated donor sites	0,028	0,024	n/s	
Mutated acceptor sites	0,022	0,023	n/s	
All	0,025	0,023	n/s	0,023
***RESCUE-ESE***				
Mutated donor sites	0,090	0,108	n/s	
Mutated acceptor sites	0,100	0,080	n/s	
All	0,091	0,094	n/s	0,099
***PESE***				
Mutated donor sites	0,048	0,082	0,007	
Mutated acceptor sites	0,057	0,055	n/s	
All	0,055	0,064	0,023	0,064
***PESS***				
Mutated donor sites	0,012	0,008	n/s	
Mutated acceptor sites	0,009	0,007	n/s	
All	0,011	0,007	n/s	0,007
**Splice site scores**				
***Mutated donor sites***				
Authentic donor sites	18,52	18,49	n/s	18,82
Acceptor sites of the (upstream) exon	18,70	19,67	n/s	19,08
Acceptor sites of the (downstream) intron	19,37	18,98	n/s	19,09
***Mutated acceptor sites***				
Authentic acceptor sites	19,59	18,72	0,05	19,08
Donor sites of the (downstream) exon	18,44	18,56	n/s	18,82
Donor sites of the (upstream) intron	18,48	18,51	n/s	18,79
**Distance to the closest candidate site(nucleotides)**				
Mutated donor sites	220,5	75	0,067	289
Mutated acceptor sites	185	66	0,024	81

The S-exons were found to be significantly shorter than the C-exons (median sizes 114 and 136). No significant differences were observed in the lengths of flanking introns (data not shown).

Scores of authentic splice sites and all splice sites in the adjacent exons and introns for the S- and C-exons were calculated as described in Data and Methods. Unexpectedly, the authentic acceptor sites affected by mutations were significantly weaker in the C-exons than in the S-exons, with the median scores 18.72 and 19.59, respectively (the Mann-Witney test P = 0.05). No significant differences were observed in the distribution of authentic site scores in the S- and C-exons with mutated donor sites, neither in the distribution of scores of all other considered sites.

The relative enrichment by potential cryptic sites near the mutated sites was estimated by calculating the distance to the closest equivalent splice site; the latter were defined as candidate splice sites of the same type as the authentic site and having the same or higher splice site score. The search for equivalent splice sites was limited to the adjacent intron and exon, and the cases when such sites were absent were not taken into account in calculations. Both for the donor and acceptor site mutations, the S- and C-exons differed dramatically: the equivalent sites were located much closer to the authentic splice sites of the C-exons than for the S-exons.

The densities of ESEfinder SF2/ASF and SRp40 motifs, as well as PESE octamers were significantly higher in the C-exons than in the S-exons with mutated donor sites, although the tendency was the same for most other types of ESEs and also in exons with mutated acceptor sites. The densities of PESS in exons with mutated splice sites of both types were higher in the S-exons, but the difference was not significant even for combined sets (The Kolmogorov-Smirnov test P = 0.09).

## Discussion

The overall results of this study seem to agree with the existing biological models. The fact that retained introns are relatively short is consistent with the possibility that such introns are spliced out by the intron definition mechanism, as in this case splicing aberrations should lead to intron retention. When this study was completed, similar observations were made also in [9].

The relative weakness of splicing sites in retained introns and the fact that exons skipped due to mutations of splice sites do not have strong cryptic sites in the immediate vicinity shows that the site scores are a reasonable approximation to site strength and may determine their functionality [3,31-33,39,40] At that, unlike [3], the relative dearth of cryptic candidate sites in the vicinity of the C-exons was not confined to exclusively to the exons with mutated donor splice sites. On the other hand, we could not confirm the observation that strong acceptor sites are a characteristic of the C-exons with mutated donor sites [31].

In contrast to previous studies that were primarily interested in functional (e.g. conserved) alternative splicing of retained introns [8,10], we did not enforce possible functionality. One of consequences of that is that the majority of retained introns studied here are unlikely to encode functional proteins, as only 3.3% of them are frame-preserving (this number is close to 4.6% in-frame retained introns observed in Arabidopsis [10]). This does not preclude the possible role of such introns in regulation, either on the protein level (e.g. leading to the synthesis of shortened proteins with regulatory function) or on the mRNA level (leading to NMD-inducing isoforms in some specific conditions); some examples of such regulatory mechanisms have been mentioned in the Introduction. However, both the procedure and the obtained results seem to indicate that the majority of retained introns in our study come from underspliced transcripts.

In line with this reasoning, the weakness of sites in retained introns may have two explanations. The retained introns might come from underspliced transcripts (weaker sites imply lower splicing efficiency) or be instances of regulated alternative splicing. Indeed, functional alternative splice sites are weaker than constitutive splice sites [41,42]. Further, longer introns in general tend to have stronger splice sites; however, the latter trend becomes observable only for bona fide introns longer than 1500 nt [43], and thus should not influence the majority of retained introns studies here.

It has been demonstrated that both human and plant retained introns are more prevalent in the 5'- and especially 3'-untranslated regions, compared to the protein-coding regions of the mRNAs mechanism [8,10]. This has been ascribed to elimination of abnormally spliced mRNAs by the NMD mechanism [44]. However, this would not explain the observed prevalence of NMD-inducing retained introns in the 5'-regions. Our results demonstrate monotonic increase in the fraction of mostly retained introns in the 5'-to-3' direction. This is consistent with some degree of co-transciptional splicing (as opposed to simple commitment to splicing with the actual process starting simultaneously for all intron) observed in experiment [45]. However, this correlation is not straightforward. Indeed, since we considered only introns bounded on both sides by internal exons, and required that the boundaries of the exon containing the unspliced intron coincided exactly with the boundaries of the corresponding exon-intron-exon chain in the RefSeq mRNA isoform (see Methods), all retained introns considered here are followed by spliced out introns. This means that the observed tendency may not be a simple consequence of completely unspliced 3'-termini.

The observed differences in the density of exonic splicing enhancers in the retained and constitutive introns as well as in the C-exons and S-exons also seem to have a natural biological interpretation. Indeed, a high density of ESE-like sites in an (relatively short) intron may lead to misrecognition of this intron as a part of an exon together with the flanking exons. Similarly, a high density of ESEs in an exon with a mutated site may force the splicing machinery to retain this exon and use a cryptic site, whereas ESSs might provoke skipping the exon. A puzzling observation that candidate enhancers predicted by RESCUE-ESE were more abundant in the constitutively splice introns than in retained ones may be explained by the fact that this method, unlike PESX, is based on the comparison of oligonucleotide frequencies in constitutive and alternative exons and does not control for the distribution of these oligonucleotides in introns [35-37]. A similar observation was recently made in [33]. Another coincidence between our study and [33] is that not all SELEX-based ESEFinder candidate exonic splicing enhancers have different densities in the S-exons and C-exons: in [33], the most pronounced effect was observed for SF2/ASF, whereas in our study a more statistically significant difference was seen for SRp40. In retained introns, the most prevalent candidate splicing enhancers were those for SF2/ASF and SC35, trailed by those for SRp40 and, marginally significant, for SRp55.

Unfortunately, at present it seems impossible to repeat these analyses with intronic splicing enhancers and silencers, since no programs for their recognition are available. A more convoluted, but still plausible explanation may be found for the observed significant difference in the strength of authentic acceptor sites of the C-exons and S-exons: an exon with a weak splice site already contains more splicing enhancers than an exon with strong sites [35,46,47], and thus it is more likely to become a C-exon if the site is disrupted by a mutation.

## Conclusion

Thus the analysis of retained introns in human cDNA, exons skipped due to mutations in splice sites and exons with cryptic sites produced results consistent with the intron definition mechanism of splicing of short introns and the model of co-transcriptional splicing. Retained introns tend to be short and contain a higher density of splicing enhancers. Skipped exons contain more candidate splicing enhancers and less silencers, compared to exons with activated cryptic sites. Skipped exons also do not have strong candidate splice sites in the vicinity of mutated ones.

## Methods

### Set of RefSeq scaffolds

Human genome (version 18, March 2006) and alignments of RefSeq genes (21.02.07) and high-throughput cDNAs (16.06.07) were downloaded from the UCSC genome browser [48]; the EST data were not used. Initially the dataset contained 25388 RefSeq mRNAs. Isoforms of alternatively spliced genes were clustered by the RefSeq gene name. To avoid redundancy in the structures of alternatively spliced genes, only the longest isoform for each such gene was retained and used as the scaffold in all further calculations. Isoform lengths were calculated for spliced mRNAs. The final set of RefSeq genes consisted of 18458 genes containing 154846 internal exons and 138777 introns between such exons. All measurements and comparisons of internal exons and introns were made according to the accepted scaffold gene structures and, in the case of mutated exons, for authentic sequences.

### Sets of mutated exons

Sets of mutated exons included only internal exons affected by single-nucleotide substitutions in splice sites (from -3 to +6 for donor sites and from -15 to +2 for acceptor sites) leading to the exon-skipping (S-exons) or cryptic site activation (C-exons). The set of C-exon was also restricted to cryptic sites located in exons and introns adjacent to the mutated site. The set of C-exons with mutations in donor splice sites was obtained from [40], and contained 42 exons. The set of C-exons with mutations in acceptor sites was obtained from the DBASS3 database [39] and contained 72 exons. The set of S-exons was collected by search of published examples of exon skipping in OMIM [49] and PubMed. The collected S exons were identified in the set of RefSeq scaffolds. The final set contained, respectively, 67 and 42 S-exons with mutations in donor and acceptor sites. The sets of donor and acceptor S-exons are available as Additional files 1 and 2 respectively.

### Sets of retained and constitutive (constitutively spliced out) introns

An intron retention event was scored if the high-throughput cDNA sequencing data contained an exon that exactly covered an exon-intron-exon chain in a RefSeq gene (Fig. 1). Such intron was called a retained intron. All other introns were considered to be constitutive introns. Since parameters of flanking exons were analyzed, only introns between internal exons from the RefSeq scaffolds were considered. The final set consisted of 1197 retained and 137580 constitutive introns.

### Splice site scores

Scores of the donor and acceptor splicing sites were calculated using positional weight matrices covering positions from -3 to +6 (for donor sites) and from -15 to +2 (for acceptor sites). The positional nucleotide weights were calculated as in [50]: W(b,m) = log [N(b,m)+0.5]-0.25·Σ_i=A,C,G,T _log [N(i,m)+0.5] where N(b,m) is the count of nucleotide b in position m in the training sample. The training sample was obtained from the EDAS database [6], and contained 4179 constitutive internal exons confirmed by at least 50 EST. The score of a donor site (b_-3_,...,b_6_), where b_j _are nucleotides, was then calculated as a sum of positional weights: S(b_-3_,...,b_6_) = W(b_-3_,-3)+...+W(b_6_,6), and similarly for scores of acceptor sites.

### Densities of cis-acting elements

Putative cis-regulatory elements were identified in all internal exons and introns by several published methods. In particular, we searched for ESE motifs initially identified by SELEX (SF2/ASF, SC35, SRp40, SRp55) using ESEfinder [34]; 238 ESE hexamers predicted by RESCUE-ESE [35]; and 2060 ESE and 1018 ESS octamers predicted by PESX [36,37]. The densities of predicted regulatory elements were defined as the number of candidate of ESE sand ESS per base pair.

### Statistical analysis

The statistical significance of differences between distributions of all intron parameters was measured by the Two sample Kolmogorov-Smirnov test and Student's t-test. The only exception was the distributions of the intron ordinal number, where we used the χ^2 ^test instead of the Kolmogorov-Smirnov test. The significance of differences between mutated exon parameters, due to small data set size was measured by the Mann-Whitney test. All these tests were implemented in the R-Package [51].

## Authors' contributions

MSG conceived the project. EZK collected and analyzed the data. MSG and EZK wrote the manuscript.

## Supplementary Material

Additional file 1 **List of skipped exons (S-exons) with mutations in donor sites**. List of skipped exons (S-exons) with mutated donor sites: gene name, ordinal number of the skipped exon in the gene, exon sequence. Click here for file

Additional file 2 **List of skipped exons (S-exons) with mutations in acceptor sites**. List of skipped exons (S-exons) with mutated acceptor sites: gene name, ordinal number of the skipped exon in the gene, exon sequence. Click here for file
